# A Case of de la Chapelle Syndrome

**DOI:** 10.7759/cureus.48150

**Published:** 2023-11-02

**Authors:** Nirja Thaker, Pratapsingh Parihar, Rajasbala Dhande, Nishant Raj, Bhavik Unadkat

**Affiliations:** 1 Department of Radiodiagnosis, Jawaharlal Nehru Medical College, Datta Meghe Institute of Higher Education and Research (Deemed to be University), Wardha, IND

**Keywords:** ambiguous genitalia, pediatric mri, de la chapelle syndrome, virilization, disorder of sex development (dsd)

## Abstract

A rare disorder of sex development (DSD) linked to a 46,XX karyotype is characterized by male external genitalia, which can range from typical to atypical, often accompanied by testosterone deficiency. A 3-year-old child who appeared phenotypically male was brought to the hospital by his parents due to concerns about ambiguous genitalia. A comprehensive series of pathological tests and radiological imaging studies were conducted to ascertain the underlying cause of his presentation. Karyotyping revealed a 46,XX genotype, while magnetic resonance imaging (MRI) results indicated the presence of both testes and a Müllerian remnant. Consequently, the diagnosis was established as the de la Chapelle syndrome. This case report aims to highlight various imaging findings associated with this syndrome.

## Introduction

The de la Chapelle syndrome, also known as 46,XX testicular disorder of sex development (DSD) [1], is an exceptionally rare genetic disorder that influences sexual development. Within this condition, individuals with a female karyotype (46,XX) experience undescended testes and ambiguous genitalia stemming from irregular gonadal and genitalia development during fetal gestation [2]. Collectively, DSD constitute a multifaceted and diverse array of conditions that can exert substantial impacts on an individual's physical, psychological, and social well-being. Enhanced comprehension, awareness, and access to appropriate care and support can alleviate the burdens associated with DSD and foster optimal health outcomes for those affected.

In approximately 90% of cases, this syndrome is attributed to the presence of the SRY gene on the Y chromosome, which is responsible for male reproductive development. Remarkably, it becomes entangled in the genetic information exchange during meiosis in the father [3]. The SRY gene is typically situated near the terminus of the Y chromosome, yet during recombination, it translocates to the X chromosome [3-5]. These disorders manifest in various forms and impact the physical, hormonal, and genetic facets of sex development. Affected children are usually raised as males and develop a male gender identity, although they may contend with undescended testes and potential infertility owing to azoospermia [6]. Structural chromosomal anomalies, including 46,XX male syndrome, represent the second most prevalent genetic cause of azoospermia after the Klinefelter syndrome [7].

DSD [1] constitute a category of congenital disorders characterized by aberrations in chromosomal, gonadal, or anatomical sex development. Globally, it is estimated that DSD affects between one in 2,000 and 4,500 newborns. However, precise prevalence figures remain elusive due to the lack of universal diagnostic standards, reporting inconsistencies, and the stigma associated with DSD in certain cultures. Estimates suggest a higher prevalence of DSD in India, ranging from one in 500 to one in 2,000 births, exceeding the global average. DSD confers a substantial disease burden due to its potential long-term implications for physical health, fertility, sexual function, and mental well-being. Individuals with DSD often necessitate sophisticated medical interventions, such as hormone therapy, surgical procedures, and ongoing health outcome monitoring. Access to adequate care may be limited in certain regions. Moreover, DSD can engender considerable emotional and psychosocial challenges, including feelings of stigma, discrimination, and social isolation. Those with DSD may grapple with gender identity, sexual orientation, relationships, and access to suitable education, employment, and healthcare resources.

The reported incidence of 46,XX male syndromes among newborns is approximately one in 20,000. In 90% of cases, the external genitalia exhibit full virilization, leading to detection post puberty, accompanied by symptoms like hypogonadism, gynecomastia, and/or infertility [1]. Clinical presentation in the remaining 10% without a Y chromosome includes hypospadias, undescended testes, or various degrees of inadequate virilization in the external genitalia (sex-determining region Y negative). Additional clinical features comprise short stature and normal mental development [8]. The de la Chapelle syndrome carries a substantial disease burden, encompassing potential infertility, challenges related to gender identity, and social stigmatization. Lifelong medical management may also be imperative for affected individuals, including hormone replacement therapy and surgical interventions.

## Case presentation

**Figure 1:**
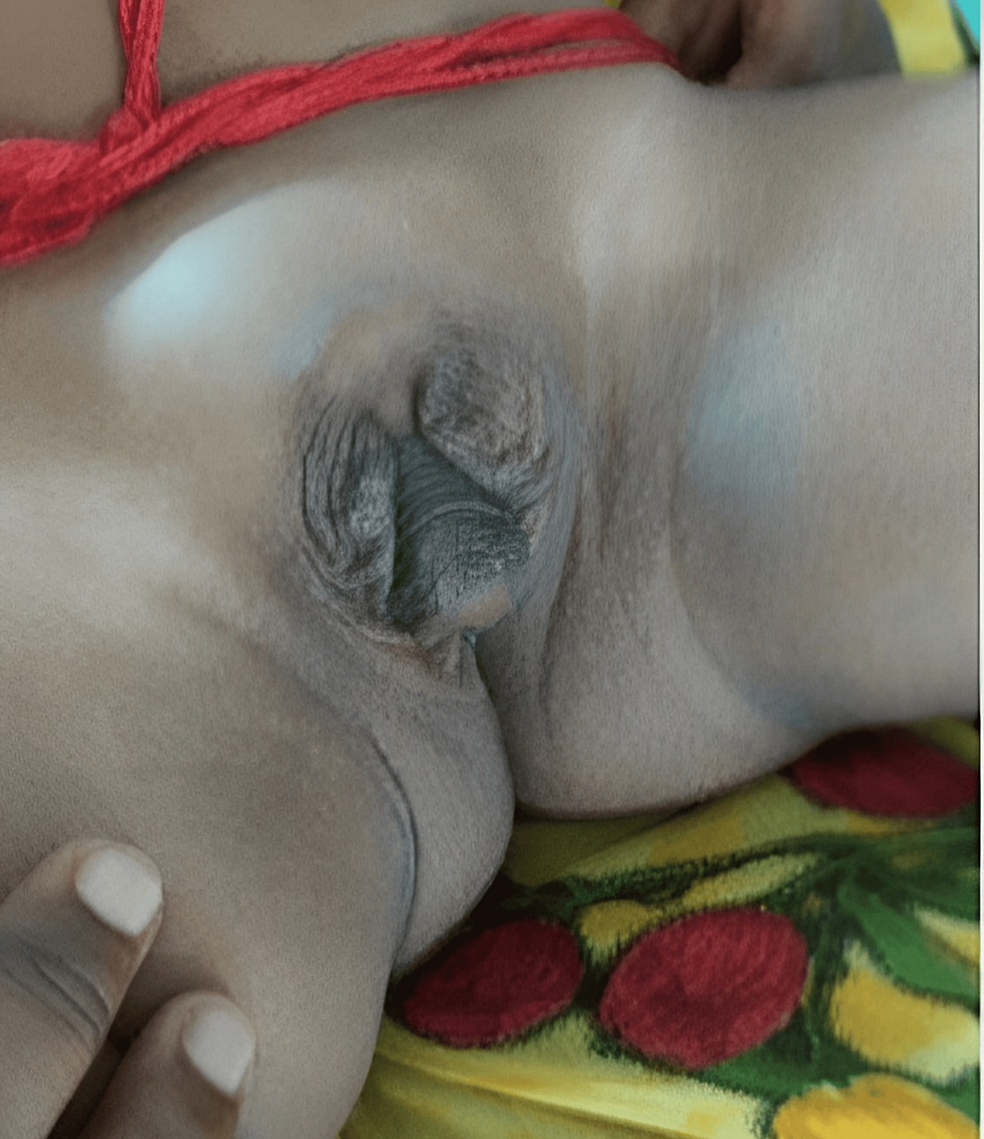
Ambiguous genitalia in the child

A 3-year-old patient, phenotypically male, received a diagnosis of undescended testes during their neonatal examination. As the child continued to grow, their father observed atypical genitalia and promptly sought medical evaluation at the hospital. Upon examination, the patient's genitalia exhibited ambiguity, as depicted in Figure 1. The patient's birth history indicates a full-term delivery with a birth weight of 1.75 kg. There is no known history of this disorder in other family members, and the mother denied using any hormones or drugs during her pregnancy.

During the laboratory investigations, the levels of dihydrotestosterone (DHT), androstenedione, and testosterone were found to fall within the established range for normalcy (DHT=22.37 ng/dl, androstenedione=0.3 ng/dl, and testosterone=4.9 ng/dl) when compared to the standard male hormone levels for that specific age group. Subsequently, a post-human chorionic gonadotropin (HCG) stimulation test indicated an elevation in testosterone levels (post-HCG stimulation: testosterone=36.5 ng/dl, DHT=86.39 ng/dl, and androstenedione=0.3 ng/dl). As seen in many cases of external genitalia ambiguity in pre-pubertal children, the levels of DHT, androstenedione, and testosterone were found to be in normal range for age. In this case also, we found them to be in normal range. The beta-HCG stimulation also revealed normal response (fourfold increase in DHT, androstenedione, and testosterone levels). Karyotyping analysis revealed a chromosomal complement of 46,XX.

**Figure 2:**
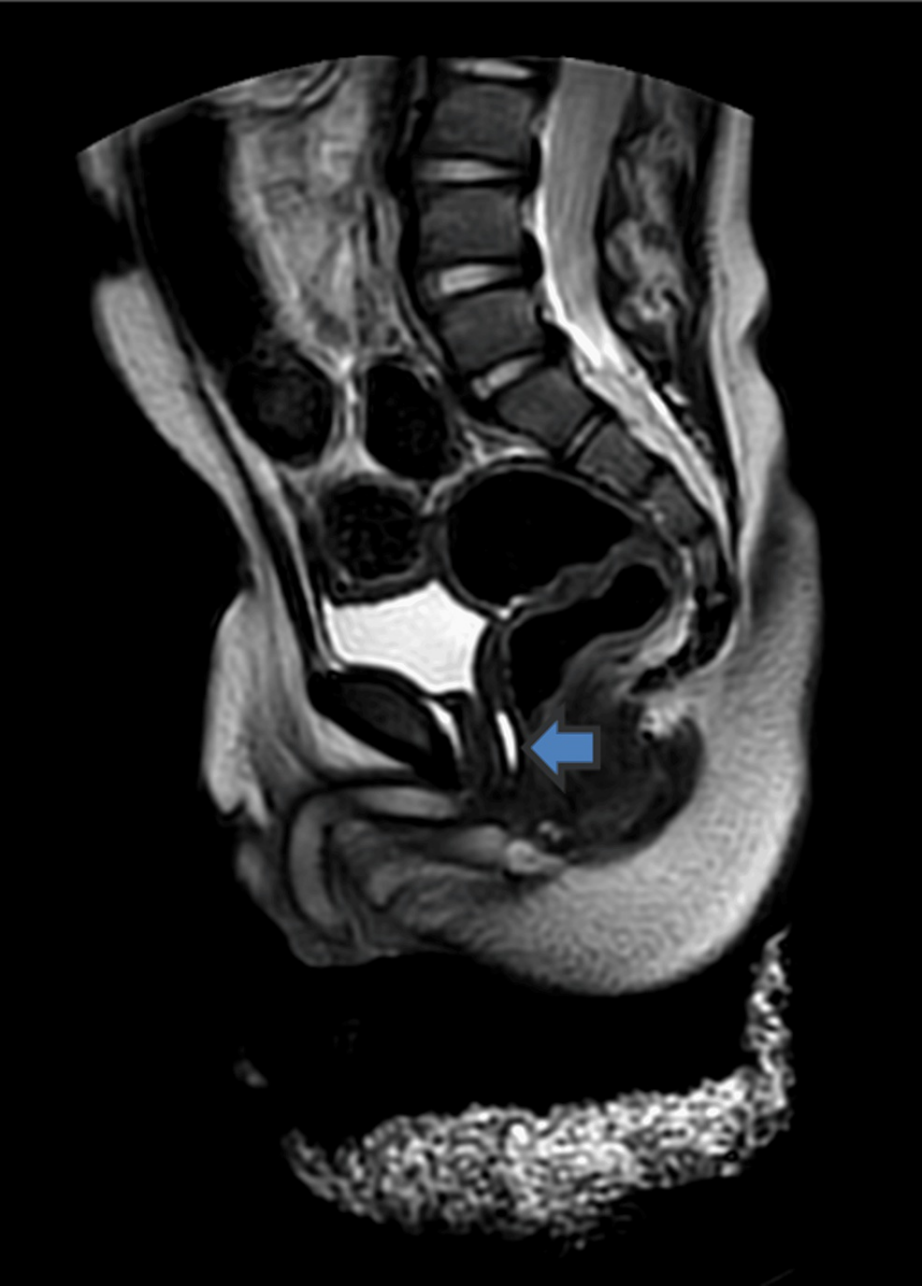
Remnant of the Müllerian duct seen as the hyperintense structure posterior to the urethra and anterior to the rectum

**Figure 3:**
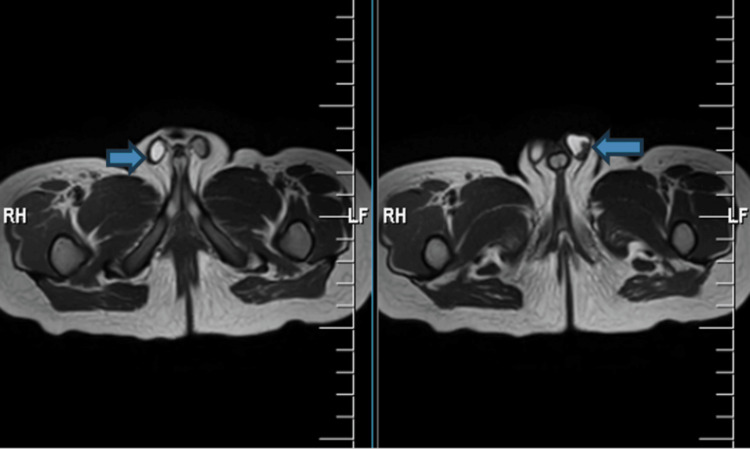
Presence of bilateral testes on the MR T2WI axial section of the pelvis MR T2WI: magnetic resonance T2-weighted image

As depicted in Figure 2 and Figure 3, bilateral testes were observed within the inguinal region. A phallus, comprising the corpus cavernosum and corpus spongiosum muscle with an inferiorly positioned urethral orifice (hypospadias), was visualized. Concentric labioscrotal folds surround the phallus. A cystic lesion was detected in the midline, anterior to the rectum, and posterior to the urinary bladder within the pelvic region. The pelvic bones, sacroiliac joints, hip joints, joint spaces, articular margins, and urinary bladder all exhibited normal appearances. The visualized spine appeared unremarkable.

The patient has been diagnosed with 46,XX testicular DSD, also referred to as the de la Chapelle syndrome [1], based on the provided information. This condition is a rare genetic disorder characterized by individuals with a female karyotype (46,XX) who exhibit undescended testes and ambiguous genitalia due to the abnormal development of the gonads and genitalia during fetal development [2].

The magnetic resonance imaging (MRI) results reveal the presence of a phallus with corpus cavernosum and corpus spongiosum, concentric labioscrotal folds surrounding the phallus, indicative of ambiguous genitalia, and an inferiorly located urethral orifice, a condition known as hypospadias. Additionally, a midline cystic lesion located anterior to the rectum and posterior to the urinary bladder in the pelvis is most likely a Müllerian duct remnant, a characteristic feature often observed in cases of 46,XX testicular DSD. Undescended testes are another distinctive symptom associated with 46,XX testicular DSD.

## Discussion

SRY-positive XX males possess two X chromosomes, one of which harbors genetic material from the Y chromosome, notably the SRY gene. This specific gene endows them with a male phenotype, despite their chromosomal configuration being more characteristic of females [3]. However, XX males lack the SRY gene (SRY negative), and in such cases, an alternative gene on one of the autosomes may instigate the development of a male phenotype. This condition arises from an erroneous exchange of genetic material between chromosomes, a process known as translocation, which occurs randomly during the development of the affected individual's father's sperm cells. The SRY gene is typically located near the terminus of the Y chromosome and can translocate to the X chromosome during recombination [3-5]. Remarkably, even without a Y chromosome, offspring from a sperm cell carrying an X chromosome with the SRY gene will develop as males. This condition is termed SRY-positive 46,XX testicular DSD [6].

The case report pertains to a patient presenting with a suspected sexual differentiation disorder, specifically identified as the de la Chapelle syndrome, characterized by the presence of a 46,XX karyotype in an individual with a male phenotype. This syndrome arises due to an error in the sexual differentiation process, leading to the formation of testes in a chromosomal female. In instances where clinical and/or laboratory symptoms of androgen deficiency manifest during puberty, testosterone replacement therapy should be administered. Additionally, for cases involving external genital anomalies, timely surgical correction is recommended to avert social and sexual challenges [1]. Although the testis morphology is typically normal in infancy, the hyalinization of seminiferous tubules in early childhood results in the loss of spermatogonia [9,10].

In this case, the patient was initially diagnosed with undescended testes during routine neonatal care. Subsequently, his father sought medical evaluation for atypical genitalia. A thorough examination revealed unambiguous genitalia; hormonal tests indicated average androgen levels. A post-HCG stimulation test confirmed adequate testosterone production, signifying functional testes. An MRI scan disclosed the presence of a phallus with corpus cavernosum and corpus spongiosum muscle, a concentric labioscrotal fold surrounding the phallus, and a linear midline cystic lesion in the pelvis, potentially a Müllerian duct remnant. In a patient presenting with male genitalia and testicular tissue, the presence of a 46,XX karyotype aligns with the de la Chapelle syndrome. A midline cystic lesion may also suggest the persistence of a Müllerian duct remnant, a common occurrence in individuals with sexual differentiation challenges.

This case underscores the imperative need for a comprehensive evaluation of patients suspected of having sexual differentiation issues, encompassing hormonal assessments, karyotyping, and imaging tests to uncover underlying genetic and anatomical abnormalities. The management of the de la Chapelle syndrome typically involves a multidisciplinary approach, incorporating endocrinologists, geneticists, and urologists. Treatment options are guided by the patient's age and degree of virilization. In cases involving ambiguous genitalia, such as the one described here, surgical intervention is usually recommended to remove any remaining Müllerian duct remnants and correct hypospadias. The specific surgical technique may vary depending on the size of the Müllerian duct remnant and the patient's future fertility desires. It may include procedures such as vaginectomy, uterine removal, and gonadectomy. Moreover, to address concerns related to gender identity and sexuality, psychological counseling and support may be deemed necessary.

The management should be centered on three primary domains: early stabilization, precise diagnosis, gender of rearing decisions, and surgical intervention and hormonal treatment planning. The fundamental aims of surgery are to achieve the best aesthetic outcomes possible, to retain sexual functioning, to preserve fertility if possible, and to reduce the risk of malignancy in the dysgenetic gonad. Delaying surgery until the child is old enough to confirm their gender identity is frequently advocated. The date of gonadectomy is determined by the risk, gender of raising, and gonad functionality. In individuals to be raised as females, removal is recommended during genitoplasty. Males should have orchidopexy and a biopsy after puberty. Streak gonads should be removed as soon as possible [11].

The surgical and medical treatment helps in improving the patient's quality of life. But infertility is usually seen in these patients. Psychosexual development is influenced by societal and cultural norms, in utero androgen exposure, genetic variations, and familial dynamics. The de la Chapelle syndrome is linked to long-term mental issues, such as low self-esteem, sadness, and so on, as well as physical complications, such as infertility. Early counseling and therapy for both the patient and his family should prevent the psychosocial influence on the individual's quality of life.

In the absence of radiological evidence of the existence or absence of testes, anti-Müllerian hormone (AMH), which acts as a marker of testicular tissue, is used to investigate ambiguous genitalia. Gonadal dysgenesis, testicular regression syndrome, disappearing testicular syndrome, and persistent Müllerian duct syndrome are potential reasons of low AMH in patients. Individuals who respond normally to beta-HCG are also likely to have androgen insensitivity syndrome, an AMH receptor deficiency, or exposure to endocrine disruptors such as phenytoin. Cloaca, urogenital sinus, rectovaginal fistula, and numerous congenital duplications such as caudal duplication are also observed in the differential diagnosis [11].

The main limitation of the study is the inability to follow-up the case. Other limitations include monetary factor (mainly for investigations like fluorescence in situ hybridization (FISH)) and social factors leading to parents hiding the patient's condition.

## Conclusions

The patient has been diagnosed with 46,XX testicular DSD, also referred to as the de la Chapelle syndrome, based on the provided information. This is a rare genetic condition characterized by individuals with the female karyotype (46,XX) exhibiting undescended testes and ambiguous genitalia due to the abnormal development of the gonads and genitalia during fetal development. The MRI results reveal the presence of a phallus with corpus cavernosum and corpus spongiosum, concentric labioscrotal folds surrounding the phallus, indicative of ambiguous genitalia, and an inferiorly positioned urethral orifice, a condition known as hypospadias. Additionally, a midline cystic lesion located anterior to the rectum and posterior to the urinary bladder in the pelvis is most likely a remnant of the Müllerian duct, commonly observed in 46,XX testicular DSD cases. Undescended testes are another distinguishing feature of this condition. The management and treatment of the de la Chapelle syndrome typically involve a multidisciplinary team of endocrinologists, geneticists, and urologists. The patient's age and degree of virilization influence the treatment choice. In the case of the patient in question, the presence of ambiguous genitalia indicates a high level of virilization. Consequently, surgical intervention is usually recommended to remove the Müllerian duct remnants and correct the hypospadias. The specific surgical approach may vary depending on the size of the Müllerian duct remnant and the patient's future fertility desires, and it may involve procedures such as vaginectomy, uterine removal, and gonadectomy. To address concerns related to gender identity and sexuality, psychological counseling and support may be necessary. The de la Chapelle syndrome is typically managed through hormone replacement therapy to promote the development of secondary sexual characteristics alongside surgical interventions to address genital anomalies. In this case, further assessment and treatment planning by an endocrinologist and a urologist may be warranted to determine the most appropriate course of action.
